# Acute Cholestatic Liver Injury Due to Ciprofloxacin in a Young Healthy Adult

**DOI:** 10.7759/cureus.13340

**Published:** 2021-02-15

**Authors:** Wiqas Ahmad, Muhammad Waqar, Muhammad Hanif Hadi, Agha Syed Muhammad, Nasir Iqbal

**Affiliations:** 1 Internal Medicine, California Institute of Behavioral Neurosciences & Psychology, Fairfield, USA; 2 Gastroenterology and Hepatology, Hayatabad Medical Complex Peshawar, Peshawar, PAK; 3 Internal Medicine, The Dudley Group NHS Foundation Trust, Dudley, GBR; 4 Internal Medicine and Gastroenterology, Russells Hall Hospital, Dudley, GBR; 5 Internal Medicine, Khyber Teaching Hospital, Peshawar, PAK

**Keywords:** drug induced liver injury, ciprofloxacin-induced cholestatic liver injury

## Abstract

Ciprofloxacin is a commonly prescribed antibiotic due to its broad spectrum and good safety profile. However, recent evidence suggests that it has the propensity to cause idiosyncratic drug-induced liver injury. There are 25 reported cases of ciprofloxacin induced severe liver injury in the literature. Here, we describe another case of acute cholestatic liver injury due to ciprofloxacin. A 32-year-old female presented to the gastroenterology department with a week's history of pruritus, jaundice, and abdominal pain. Her symptoms started three days after completing a ciprofloxacin course for urinary tract infection. Her hepatic enzymes were elevated and showed a cholestatic pattern. An extensive workup, including viral serology, autoimmune profile, and imaging studies, did not reveal any underlying cholestasis cause. Her liver biopsy findings were consistent with drug-induced cholestasis.

A diagnosis of ciprofloxacin-induced cholestatic liver injury was made based on the onset of symptoms and liver enzyme derangements following the use of ciprofloxacin, improvement in clinical as well as biochemical parameters after cessation of ciprofloxacin, and the liver biopsy findings. The patient received supportive treatment, and her liver enzymes returned to baseline six weeks after admission. Clinicians need to be aware that if the patient develops any liver injury symptoms while using ciprofloxacin, the drug should be stopped immediately, and a thorough evaluation should be done. The patient should also be advised to avoid ciprofloxacin and other quinolones in the future.

## Introduction

Cholestatic drug-induced liver injury is one of the most severe manifestations of drug-induced liver injury (DILI), and establishing its diagnosis can be challenging due to variable clinical features, multiple differential diagnoses, and unavailability of specific diagnostic tests. Cholestatic DILI is associated with a high mortality rate of 10%; therefore, immediate recognition and the causative agent's withdrawal are indispensable [1, 2]. Most cases of DILI can be attributed to antibiotics and anticonvulsants [3].

Ciprofloxacin is a fluoroquinolone antibiotic with broad antimicrobial coverage that is usually well-tolerated and has a good safety profile [4]. It usually causes mild, transient elevation of liver enzymes in 2 to 3% of patients [5]. However, now evidence is emerging suggesting that ciprofloxacin can cause an idiosyncratic form of DILI. Till now, 25 cases of idiosyncratic DILI due to ciprofloxacin have been reported in the medical literature [4]. Herein, we describe another case of acute cholestatic hepatitis due to ciprofloxacin.

## Case presentation

A 32-year-old, married female was referred to our gastroenterology department with a week's history of pruritus, jaundice, and abdominal pain. Her medical history was remarkable for recurrent urinary tract infections, treated with various antibiotics at different intervals. She had received fosfomycin and cefixime in the past, however, these antibiotic courses were taken three months prior to the onset of these symptoms. Two weeks before the onset of these symptoms, she was commenced on a 10-day course of ciprofloxacin for her urinary tract infection. On the third day following the antibiotic course completion, she developed these symptoms.

**Figure 1:**
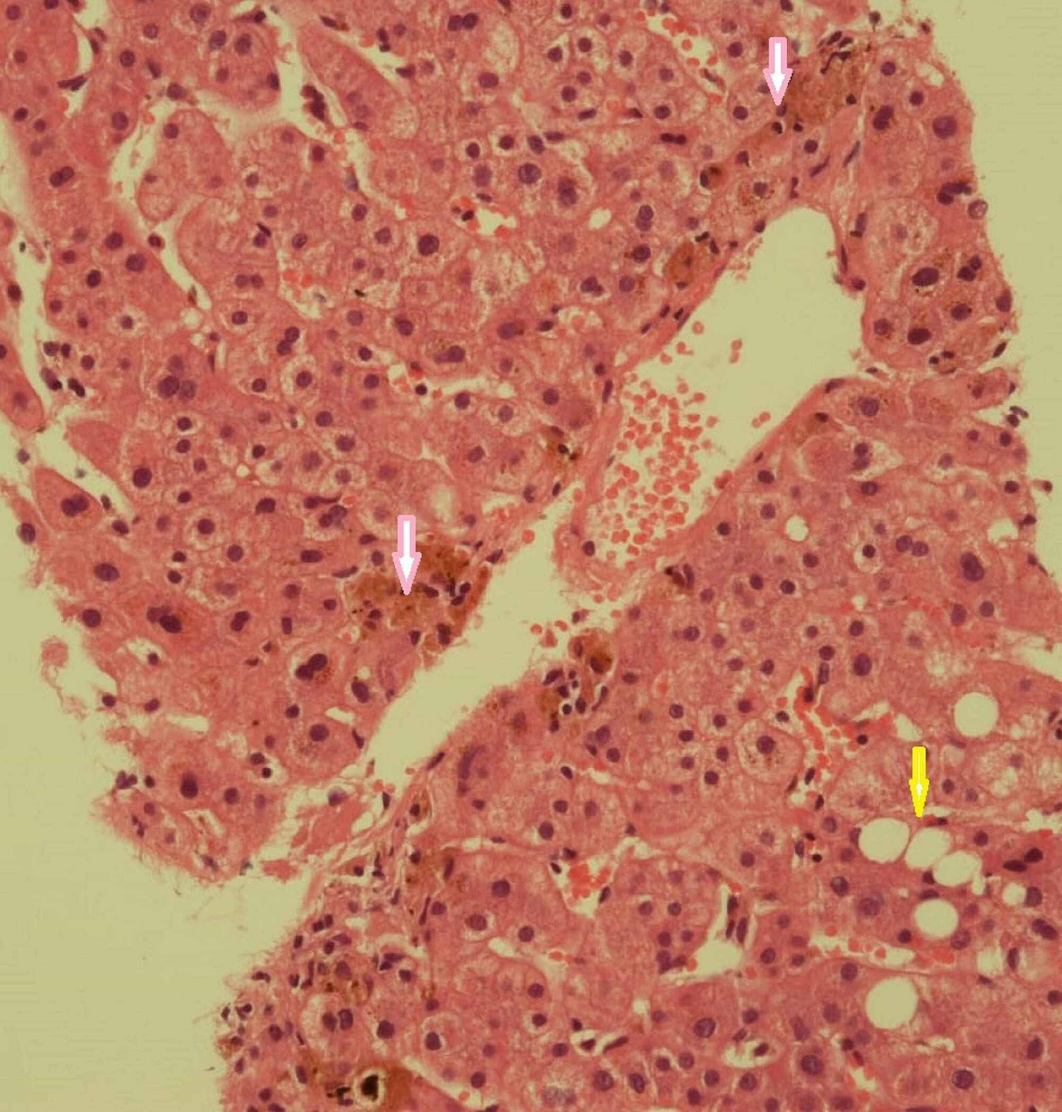
Histological section of the liver shows canalicular and hepatocellular cholestasis (pink arrows) and macrovesicular steatosis (yellow arrow)

On examination, she was deeply jaundiced and had widespread excoriations, especially involving the upper and lower limbs. Her investigations done on arrival were as follows; total bilirubin was 16.7mg/dL (reference range: 0.1 - 1 mg/dL), direct bilirubin 13.3mg/dL (reference range: 0.0 -0.3 mg/dL), indirect bilirubin 3.4mg/dL (reference range: 0.2 -0.8 mg/dL), alanine transaminase (ALT) 172 U/L (reference range: 5 -35 U/L), alkaline phosphatase (ALP) 866 U/L ( reference range: 30 -129 U/L); however, complete blood count, renal functions and serum electrolytes were in the normal range. Her ultrasound abdomen revealed sludge in the gall bladder; the common bile duct was 6 mm in diameter. A subsequent contrast-enhanced computed tomography (CT) scan of the abdomen was reported normal; therefore, a magnetic resonance cholangiopancreatography ( MRCP) was performed, which revealed gall bladder sludge and a simple hepatic cyst; however, the biliary tree was normal. Serology of hepatitis A, B, C, E virus, anti-nuclear antibodies, anti-mitochondrial antibodies, anti-smooth muscle antibodies, and serum IgG-4 was negative. The case was discussed in the hospital multi-disciplinary team meeting, and a decision of liver biopsy was taken in view of her slow initial improvement and to ascertain the cause of cholestasis, it revealed centrivenular cholestasis and focal mixed steatosis, fibrosis score 0/6, favoring drug-induced cholestasis (Figure 1)

A diagnosis of ciprofloxacin-induced cholestatic liver injury was made after excluding other causes of cholestasis. Though the patient used other antibiotics such as fosfomycin and cefixime in the past for her urinary tract infection, they were used three months prior to the onset of these symptoms, therefore it is least likely that they may have contributed to this liver injury. The onset of symptoms and liver enzyme derangements following the use of ciprofloxacin, improvement in clinical as well as biochemical parameters after cessation of ciprofloxacin, and the liver biopsy findings all were suggestive of ciprofloxacin induced cholestatic liver injury. She remained under our care for two weeks, and supportive treatment was commenced. Initially, after one-week little improvement in liver function tests (LFTs) was noted, but from there onwards, her LFTs started to improve, and after six weeks, her LFTs normalized. The Roussell Uclaf Causality Assessment Method (RUCAM), a structured assessment scale used to estimate the probability of liver injury due to a particular drug, showed that this patient had a scale classified as "highly probable".

 Table 1 summarizes the trend of her LFTs and international normalized ratio (INR) over the passage of time.

**Table 1:** Shows the trend of patient's LFTs and INR LFTS: Liver function tests, ALT: Alanine aminotransferase, ALP: Alkaline phosphatase, INR: International normalized ratio

LFTs and INR (normal range)	On admission	1week after admission	2weeks after admission	4weeks after admission	6weeks after admission
Bilirubin (0 - 1mg/dL)	16.7	15.8	9.7	3.2	0.9
ALP (30 - 129 U/L)	866	778	345	236	87
ALT (5 - 35 U/L)	172	154	76	49	28
INR (0.9 - 1.1)	1.6	1.4	1.2	1	1

## Discussion

Drug-induced liver injury (DILI) can be further classified into two types: Intrinsic DILI and Idiosyncratic DILI. Intrinsic DILI is more common and depends on the dose of the drug administered, while idiosyncratic DILI is less frequent and occurs independently of the administered drug dose, and may have different clinical presentations [6, 7]. According to the available clinicopathological evidence, ciprofloxacin can cause dose-independent idiosyncratic DILI. The idiosyncratic nature of ciprofloxacin-induced DILI is substantiated by the sudden onset of injury and the lack of association between ciprofloxacin dose and the onset and severity of injury [4]. Patients with DILI usually present with nonspecific symptoms such as lethargy, nausea, and abdominal pain; however, it may manifest with more severe symptoms like jaundice, ascites, and coagulopathy encephalopathy [3].

Most ciprofloxacin-induced liver injury cases manifest within two to three weeks after the initiation of therapy [4]. The exact mechanism by which ciprofloxacin causes liver injury is yet unknown, however hepatocellular necrosis resulting in raised liver enzymes has been observed [8]. The ciprofloxacin-induced liver injury usually presents with a hepatocellular pattern or cholestatic pattern of liver function tests (LFTs), although rarely mixed pattern, has also been reported [9]. 

Diagnosis of DILI is difficult as it is often a diagnosis of exclusion, and additionally, there are no specific biomarkers for most of the drugs that cause liver injury [10]. Therefore a high index of suspicion is required to diagnose DILI. Additionally, if the patient develops any symptoms or has abnormal liver function tests while using ciprofloxacin, the drug should be stopped immediately, and thorough evaluation should be done. Although liver biopsy is not required in all suspected DILI cases [4], it was performed in the current case because the patient's biochemical parameters initially showed little improvement despite stopping the offending drug.

Prompt withdrawal of the offending drug and supportive care are considered the mainstay of treatment in cases of DILI. Liver enzymes should be monitored regularly until they become normal [4, 9].

Drug-induced icteric hepatocellular injury is associated with higher mortality compared to cholestatic DILI, which has a more benign and self-limiting course [10]. The prognosis of ciprofloxacin-induced liver injury is good. Apart from the three cases of fulminant hepatitis and death, the remainder of the reported cases of ciprofloxacin-induced liver injury improved rapidly after its cessation [3]. In our case, the patient showed improvement two weeks after cessation of ciprofloxacin and achieved complete recovery six weeks after admission.

Ciprofloxacin belongs to the quinolone group; therefore if the patient develops ciprofloxacin-induced DILI, the patient should be advised to avoid not only ciprofloxacin but other quinolones also due to cross-sensitivity [8].

## Conclusions

Although ciprofloxacin has a good safety profile, it has the propensity to cause severe drug-induced liver injury and, in very rare cases, acute liver failure. Therefore, in suspected ciprofloxacin-induced liver injury, prompt recognition and immediate withdrawal of ciprofloxacin are necessary to ensure a smooth recovery and prevent mortality. In addition, patients with ciprofloxacin-induced liver injury should be advised to avoid ciprofloxacin and quinolone antibiotics in the future.

## References

[1] Gijbels E, Vinken M (2019). Mechanisms of drug-induced cholestasis. Methods Mol Biol.

[2] Sundaram V, Björnsson ES (2017). Drug-induced cholestasis. Hepatol Commun.

[3] Yamashita YI, Imai K, Mima K, Nakagawa S, Hashimoto D, Chikamoto A, Baba H (2017). Idiosyncratic drug-induced liver injury: a short review. Hepatol Commun.

[4] Radovanovic M, Dushenkovska T, Cvorovic I (2018). Idiosyncratic drug-induced liver injury due to ciprofloxacin: a report of two cases and review of the literature. Am J Case Rep.

[5] Wolfson JS, Hooper DC (1991). Overview of fluoroquinolone safety. Am J Med.

[6] Chalasani NP, Hayashi PH, Bonkovsky HL (2014). Acg clinical guideline: the diagnosis and management of idiosyncratic drug-induced liver injury. Am J Gastroenterol.

[7] Andrade RJ, Tulkens PM (2011). Hepatic safety of antibiotics used in primary care. J Antimicrob Chemother.

[8] Baloch ZQ, Raza MA, Abbas SA (2017). Ciprofloxacin-induced hepatotoxicity in a healthy young adult. Cureus.

[9] Zimpfer A, Propst A, Mikuz G (2004). Ciprofloxacin-induced acute liver injury: case report and review of literature. Virchows Arch.

[10] Hoofnagle JH, Björnsson ES (2019). Drug-induced liver injury - types and phenotypes. N Engl J Med.

